# Peak Oxygen Uptake Responses to Training in Obese Adolescents: A Multilevel Allometric Framework to Partition the Influence of Body Size and Maturity Status

**DOI:** 10.1155/2013/618595

**Published:** 2013-07-15

**Authors:** Humberto M. Carvalho, Gerusa E. Milano, Wendell A. Lopes, António J. Figueiredo, Rosana B. Radominski, Neiva Leite

**Affiliations:** ^1^Faculty of Sport Sciences and Physical Education, University of Coimbra, 3040-156 Coimbra, Portugal; ^2^Department of Physical Education, Federal University of Paraná, 81690-100 Curitiba, PR, Brazil; ^3^Department of Physical Education, State University of Campinas, 13083-851 Campinas, SP, Brazil; ^4^Pediatric Endocrinology Unit, Department of Nutrition, Federal University of Paraná, 80210-170 Curitiba, PR, Brazil

## Abstract

The influence of body size and maturation on the responses in peak oxygen uptake (VO_2_) to a 12-week aerobic training and nutritional intervention in obese boys (*n* = 30; 10–16 years) was examined using multilevel allometric regressions. Anthropometry, sexual maturity status, peak VO_2_, and body composition were measured pre- and postintervention. Significant decrements for body mass, body mass index *z*-score, and waist circumference and increments for stature, fat-free mass, and peak oxygen uptake were observed after intervention. Partitioning body size on peak VO_2_, the responses of the individuals to training were positive (11.8% to 12.7% for body mass; 7.6% to 8.1% for fat-free mass). Body mass and fat-free mass were found as significant explanatory variables, with an additional positive effect for chronological. The allometric coefficients (*k*′) in the initial models were *k*′ = 0.883 and *k*′ = 1.058 for body mass and fat-free mass, respectively. The coefficients decreased when age was considered (*k*′ = 0.530 for body mass; *k*′ = 0.860 for fat-free mass). Including maturity indicator in the models was not significant, thus the influence of variability in sexual maturity status in responses to exercise-based intervention in peak VO_2_ may be mediated by the changes in body dimensions.

## 1. Introduction

Peak oxygen uptake (VO_2_), that is, the highest oxygen uptake elicited during an exercise test to exhaustion, is recognized as the best single indicator of young people's aerobic fitness [1]. It is used in the prescription of aerobic exercises, in the monitoring of physical training adaptations, and as a predictive parameter of associated morbidities [2]. To determine the extent to which the physiological markers of aerobic fitness change as result of exposition to regular endurance exercise in children and adolescents, it is required longitudinal studies, as well as to consider the influence of age and growth during pubertal development [3]. Also, it has been extensively noted that peak VO_2_ is highly correlated with body size, thus concurrent effects of size with age and maturation on peak VO_2_ need to be appropriately accounted for [4].

The pitfalls of the use of ratio standards to partition the influence of body size in performance variables have long time pointed out [5]. Allometric techniques to partition the effects of size on performance have been found to have less bias [6]. On the other hand, the use of allometry to interpret the longitudinal data, such as responses to training, is not straightforward and may provide an incomplete interpretation of the performance measured under investigation [4].

Another analytical problem resides in the limitations of traditional approaches to analyze repeated-measures data in physiological research, in particular repeated measures ANOVA [7, 8]. One of the restrictive assumptions that may limit the models in physiological indicators interpretation is the requirement that correlations among measurements on the same subject satisfy a restrictive condition called sphericity, that is, equal variability of the measurements at each time point and equal correlations between every two measurements on the same individual [7]. An appropriate method of analyzing repeated-measures data, with the flexibility to identify and separate the relative contribution of confounding factors and allowing the use of realistic yet parsimonious variance and correlation patterns for particular applications, is to adopt this multilevel modeling approach [7, 9]. This approach is an extension of ordinary multiple regression, where the data have a hierarchical or clustered structure. In addition to describing the population mean response, the method recognizes and describes variation around the mean at both levels; for example, at level 2, individuals have their own growth rates in response to a stimulus that vary randomly around the underlying population response, and, at level 1, each individual's observed measurements may vary around their own growth trajectory.

Childhood obesity continues to be a major focus of public health efforts [10]. Also, there may be both short-term health consequences [11] and long-term tracking of obesity to adulthood [12]. Hence, there is a great interest on exercise-based interventions for health related benefits. Generally, it is aimed to educate youth on subjects of health and nutrition and promote active living, and have been proposed as attractive strategies for encouraging weight loss and reverting/preventing metabolic alterations in children and adolescents [13, 14]. Important elements of such interventions include the maintenance of fat-free mass and the improvement of functional and physical capacities for the purpose of maintaining/enhancing metabolism rates and preventing fat-mass regains [15]. The benefits of such interventions in terms of reducing body mass and visceral adiposity reduction have been well documented in youth [16].

A disproportionate enlargement with bigger individuals is obvious, both in shape and composition and in particular in obese children and adolescents. The interpretation of changes in peak VO_2_ in obese adolescents' individuals may be affected by the excess body fat need to consider appropriate analytical approaches. Thus, the purpose of this study was to model the changes in peak VO_2_-body size relations in obese boys (10–16 years) exposed to a 12 weeks endurance exercise-based intervention from an allometric perspective, and whether changes induced by training where affected by chronological age and maturity status.

## 2. Methods

Thirty obese boys, with body mass index (BMI) above age-specific 95th percentile according to the World Health Organization (WHO) [17] and the International Obesity Task Force (IOTF) [18] and aged between 10–16 years old volunteered to participate in this study. The obese boys were recruited from the Pediatric Endocrinology Ambulatory and Public Schools of Curitiba, Paraná (Brazil). All participants were physically inactive. Curitiba is an urban city located in southern Brazil, colonized mainly by European immigrants. The inclusion criteria were children or adolescent that had obesity according to the WHO and IOTF [17, 18]. Exclusion criteria were presence of diabetes and the use of medication that could alter BP, glucose, or lipid metabolism; previous drug used such glucocorticoids, insulin sensitizers, or psychotropics, which may affect appetite regulation; orthopedic limitations. Information about the frequency and duration of physical activities was retrieved and recorded using a questionnaire [19]. The study was conducted in accordance with Human Ethics Committee of the Federal University of Paraná (Protocol number CEP/HC 765.184/2003-11). All participants and their families received information about the protocol and signed an informed consent form.

### 2.1. Maturity Status

The stage of pubic hair (PH) development was assessed at clinical examination by a physician experienced in the protocol described by Tanner [20]. It provides an estimate of maturity status because it is not known when a boy entered a particular stage or how long he has been in a stage. Given the sample size and for multilevel regression analysis, boys were grouped as prepubertal group if classified as PH stage 1 or 2 (*n* = 16, age = 11.68 ± 1.14 years) and as pubescent/postpubescent group if classified as PH stage 3, 4, or 5 (*n* = 14, age = 13.45 ± 1.83 years). Replicate assessments of pubertal stage were not possible.

### 2.2. Anthropometry and Body Composition

All measurements were taken by a single experienced observer following standardized procedures [21]. Stature was measured with a portable stadiometer (Ayrton Corporation, Prior Lake, MN, USA) to the nearest 0.1 cm. Body mass was measured with a portable balance (Filizola, São Paulo, Brazil) to the nearest 0.1 kg. BMI was calculated from body mass (kg) by stature squared (m^2^). Because the amount of body fat varies for children by age and sex, BMI *z*-scores were derived against US CDC 2000 reference [22] to provide a standard indicator of relative adiposity. Waist circumference was measured in centimeters using a nonextendable flexible tape to the nearest 0.1 cm. The tape was applied above the iliac crest, parallel to the ground, with the individual standing with the abdomen relaxed, arms along the body, and feet together. Body composition was determined by bioelectrical impedance using a tetra polar machine (Biodynamics). Measurements were performed in the morning, after a 10 to 12-hour-overnight fasting. Fat-free mass, fat mass, and the percentage of body fat mass were calculated with validated equations [23] and in accordance with the National Institutes of Health guidelines [24].

### 2.3. Aerobic Fitness (Peak VO_2_)

Cardiovascular fitness was measured on treadmill using a protocol adapted from Balke [25]. Verbal encouragement was provided throughout the task. Peak VO_2_ and ventilatory responses were measured using a calibrated breath-by-breath monitoring system (Parvo Medics, True Max 2400, UT, USA), and maximal exercise heart rate was also determined (Polar-S625X, Kempele, Finland). To ensure that participants had achieved peak VO_2_, at least one of the following criteria was met: volitional exhaustion; respiratory exchange rate equal to or greater than 1.10; heart rate reached a value within 10% of the age predicted maximal heart rate; and a plateau in oxygen consumption, despite increased exercise intensity.

### 2.4. Intervention Design

The intervention consisted of aerobic exercise, a nutritional program, and educational meetings for the management of obesity. The group involved in the delivery of the program included a physiologist, a nutritionist, six physical educators, two physicians, and three nurses. The intervention took place in a clinic in the central region of Curitiba-Pr. Only the walks were made in a square nearby.

#### 2.4.1. Exercise

All participants were required to attend exercise sessions from two to three times per week during three months or 12 weeks, totalized 200 to 300 minutes per week. The weekly volume of exercise was adopted following the American College of Sports Medicine (ACSM) guidelines for obese exercise interventions [2]. Exercise consisted of 45 minutes indoor cycling, 45 minutes outdoor walking/running, and 20 minutes stretching, which were conducted by a certified fitness instructor. Exercise was prescribed for each participant, based on the data collected from the baseline treadmill and cycle ergometer test. During the first four weeks, intensity was set as 35–55% of heart rate reserve, light to moderate intensity, and was increased to 55–75% during the final eight weeks, moderate to hard intensity. Heart rate was monitored and registered every 15 minutes during all sessions. Instructors encouraged and helped participants to increase exercise intensity to maintain the heart rate within the target zones [2].

#### 2.4.2. Nutritional Intervention and Educational Meetings

The nutritional intervention was involved qualitative and quantitative analyses of the child's food intake, based on a 3 day food record, two days per week, and one day in weekend. The participant's diet control (dietary recall) was taken in three days, according to the American Dietetic Association [26]. The diet emphasized the consumption of abundant vegetables, fresh fruit, regular consumption of dairy products (principally cheese and yogurt), fish and poultry consumed in low to moderate amounts, and a reduced intake of red meat. This diet has been specifically devised for children by our nutritionist. Total fat in this diet is from 25% to 35% of the total caloric intake. Families were provided with additional nutritional instruction, including interpretation of food labels and shopping, and were taught stimulus control to reduce access to high-calorie foods and increase access to healthy lower-calorie foods. All participants received an orientation encouraging them to maintain an active lifestyle during and after the program multidisciplinary. The adolescents participated in educational meetings once-per month during 60 minutes. The meeting were taught by physical educators and nutritionist focused on daily diet and physical activities.

### 2.5. Statistical Analysis

Descriptive statistics for all measures are presented as mean ± standard deviation. Changes in body size and composition and peak VO_2_ as a consequence of training, were examined based on a two-level growth model. At level 1, each participant's successive measurements over time were defined by an individual changes with training and random error. Individual changes were expressed as percent changes (dependent variables were log-transformed). At level 2, differences in trajectories between groups of individuals were examined. To make inferences about the true (population) values of the effect of training on body size and composition and peak oxygen uptake, the 95% confidence limit for each effect was also calculated (CL) [27]. Interindividual (static) allometric coefficients (*k*) were calculated for peak VO_2_ and body mass at pretraining and posttraining. Coefficients reflect the dimensional relationship between peak VO_2_ and body dimensions at pre- and posttraining as follows:

(1) $$\begin{array}{c} Y=a\cdot X^{k}\cdot \varepsilon , \end{array}$$

where *a* is the intercept of the regression line on the *Y* axis and *k* is the slope of the line. Values of *a* and *k* were derived from linear regressions of the logarithmic regression transformations:

(2) $$\begin{array}{c} \text{Log}\;Y=\text{Log}\;a+k\cdot \text{Log}\;X+\text{Log}\;\varepsilon , \end{array}$$

where *Y* was the dependent variable (natural logarithms, i.e., log peak VO_2_) and body size (i.e., log body mass and log fat-free mass).

Multilevel regression analyses, adopting proportional curvilinear models [9], were used to examine the individual relations between peak oxygen uptake and body size in response to 12-week training in obese boys, controlling the influence of interindividual variation in age and interaction term between age and sexual maturity status (prepubescent group and pubescent/postpubescent group):

(3) Peak  VO2 =size  descriptorkij ·exp⁡(aj+bij·time  of  measurement+cj·age  or age  or  interaction  term  age  x  maturity)·εij.

Subscripts *i* and *j* denote random variations at levels 1 and 2, respectively. This model can be linearised with a log transformation, and multiple linear regressions were used to fit the unknown parameters. The log-transformed version of (3) becomes:

(4) Log(Peak  VO2) =kij·Log(size  descriptor)+aj+bij·time  of  measurement+cj·age  or  interaction  term  age  x  maturity+Log  εij.

All parameters were fixed with the exception of the constant (intercept term) and time of measurement (binary variable—pretraining coded 0, post training coded 1) parameters, which were allowed to vary randomly at level 2 (between individuals). The effect of age and interaction term between age and maturity status were added as predictors to the two-level growth model and tested in separate models to explore the influence of age and maturity status (residuals of age centered by maturity status group) on the individual changes with training (time of measurement) and body size exponents.

## 3. Results

The distribution of obese boys participating in the present study was as follows: PH1, 11 boys (average age: 11.7 ± 1.3 years); PH2, 5 boys (average age: 11.7 ± 0.6 years); PH3, 6 boys (average age: 12.7 ± 1.6 years); PH4, 7 boys (average age: 13.9 ± 1.9 years); PH5, 1 boy (age: 14.8 years). The average age and standard deviation were 11.7 ± 1.1 years and 13.4 ± 1.8 years for the prepubertal group (PH1 and PH2, *n* = 16) and pubertal/postpubertal group (PH3, PH4, and PH5, *n* = 14), respectively. As expected the boys classified in later stages of sexual maturity tend to be older than boys classified in the early stages of pubic hair development. Also the variability of chronological age in the total sample and in the obese boys grouped by PH status was substantial.

The mean changes in the variables of interest following the 12 weeks of multidisciplinary intervention in adolescent obese boys are presented in Table 1. Although significant changes in body size and free-fat mass appear to be trivial. We observed significant and possible beneficial decreases in BMI, BMI *z*-score, waist circumference, and fat mass, suggesting a likely positive effect of the intervention in fat distribution and body shape. Changes in peak VO_2_ with training were positive, likely beneficial effect.

Interindividual allometry coefficients (*k*) derived from the linear regression of the logarithmic transformation of peak VO_2_ and body dimensions (body mass and fat-free mass, see Table 2) were higher pretraining (*k* = 1.015, 95% CL 0.670 to 1.361 for body mass; *k* = 1.107, 95% CL 0.841 to 1.373 for fat-free mass) compared to exponent values posttraining (*k* = 0.817, 95% CL 0.480 to 1.154 for body mass; *k* = 1.046, 95% CL 0.811 to 1.281 for fat-free mass).

The results of the multilevel regressions including separate models for body dimensions (body mass and fat-free mass), age, and interaction term of age and maturity status as explanatory factors of change in peak VO_2_ and its relation with body dimensions are summarized in Table 3 (for body mass) and Table 4 (for fat-free mass). Since the dependent variables were log-transformed and proportional, multiplicative models were adopted, and fixed effects are interpreted as allometric exponents, that is, proportional relations with the dependent variable. Both body mass and fat-free mass proved to be significant covariates of peak VO_2_ responses to the 12-week intervention. Partitioning the influence of body mass, the changes with training in peak VO_2_ were positive and slightly higher (11.8% to 12.7%) than when considering changes in absolute peak VO_2_ (10.4%, Table 1). However, when we considered the relation between peak VO_2_ and fat-free mass, the changes with training in peak VO_2_ remained positive but more moderate (7.6% to 8.1%). Only the inclusion of age in the models was significant. Age as explanatory variable appears to have greater influence on the relationship between peak VO_2_ and body dimensions, rather than in the change with training in peak VO_2_. Interpretation of the exponents for age suggests that older obese adolescents had higher initial values of peak VO_2_, partitioning the influence of body dimensions. The allometric coefficients in models 1 were *k*′ = 0.883 (SEE = 0.148) and *k*′ = 1.058 (SEE = 0.096) for body mass and fat-free mass, respectively. When chronological age was added to the model, the size coefficients decreased to *k*′ = 0.530 (SEE = 0.158) for body mass and *k*′ = 0.860 (SEE = 0.136) for fat-free mass. At level 1, the residual variance summarizes the population variability in the average individual's change estimates around his own true change trajectory [28]. For the models considering body mass as scaling variable, the results showed significant variation at level 1 when chronological age was included in the fixed part. When fat-free mass was included as scaling variable, all models showed significant variation at level 1. Regarding variation at level 2, all models presented significant portion of variation between individuals remaining to be explained at the intercept, that is, pretraining status. However, the individual changes with training, that is, the slope, showed no between-subject variation.

## 4. Discussion

The results of the present study show that obese adolescent boys likely have beneficial effects on cardiorespiratory fitness in response to a 12-week training multidisciplinary program, accounting within an allometric framework the expected concurrent changes in body size due to growth and training.

As expected the boys stature increased during intervention. Overall, changes in body mass and composition with training were in concordance with improvements in aerobic fitness; however, only changes in BMI, BMI *z*-score, waist circumference, and fat mass appear to represent a substantial, and perhaps beneficial, difference. Reduction in the BMI *z*-score was 21.5%. Reductions in waist circumference with training were about 3.6%. The body mass loss (approximately 0.16 kg/week) and reduction of BMI were lower than that obtained in others studies with adolescent obese populations [13, 14, 29]. The results suggest that the 12-week multicomponent approach based on exercise appears to be effective to reduce visceral and abdominal subcutaneous adipose tissue, consistent with trend observed in previous data with larger exercise exposure (4 months) in 7 to 11 years old obese children [30].

In the present study, fat mass had a significant decrease and fat-free mass had a slight increase in boys with the 12-week intervention, although nonsignificant. Thus, the intensity and volume of the exercise and nutritional guidelines appear to be sufficient to maintain fat-free mass in individuals engaged in weight loss programs. The results are consistent with the positive influence of regular physical activity in adipose tissue metabolism. Nevertheless, interpretation of changes with training during pubertal development needs care as adolescents experience marked changes in body size, composition, and physique, especially those that mature in advance of their peers [31]. Particularly adolescent males experience gain in absolute fat mass; however, superior gains in absolute muscle mass generally result in a decrease in relative fat-mass during this period. This is a positive aspect given that, generally, weight loss programs with more restrictive nutritional guidelines result in significant reductions in fat-free mass [29]. Also, changes in frequency, intensity, type, or duration in the exercise interventions may increase the gains in fat-free mass; however, it would be important to balance these potential gains with the feasibility and practicality of a more vigorous intervention.

The average peak VO_2_ values in the present study falls well within the range of values reported for different populations age matched [1]. Although available data in the literature is limited, the present results are consistent with previous observations comparing VO_2_ responses between obese and nonobese adolescent boys [32].

Despite theoretical and statistical limitations noted [5], peak VO_2_ continues to be routinely expressed as a ratio standard (e.g., mL·min^−1^·kg^−1^). Interindividual allometric scaling (log-linear regression) techniques were adopted in the present study to examine variation in the peak VO_2_-body size relationship with exercise-based intervention in obese boys. The validity of the allometric models was examined by inspecting residuals of the allometric models against the respective body dimensions. No residual correlation with the respective body dimension variables was present, indicating that the models used provided peak VO_2_ “size free scores” for each of the size variables (data not presented). Surprisingly, the available data to compare size exponents in relation to peak VO_2_ in obese adolescent males is very limited, mostly using least-squares regression standards [32], despite disproportionate size and shape with obesity. Allowing for variation in protocols and instruments, allometry coefficients derived from the linear regression (Table 2) are higher to those for a sample of active boys followed from 8 to 16 years [33]. This suggests that, despite comparable absolute peak VO_2_ values with nonobese boys, the cardiac output and uptake of oxygen at tissue level is less efficient in proportion to total mass and active fat-free mass. Noteworthy, the size coefficients decreased with training indicating that the oxygen metabolism in proportion to body size (body mass or fat-free mass) during exercise becomes efficient in obese adolescents with 12-week endurance-based training.

The adoption of multiplicative allometric models within a multilevel structure allows examining levels of variability accounting for a single individual's departure from their fitted change with training trajectory and the underlying population response, plus accounting for confounding effects. It also allows each individual in the sample to have his own individual body mass exponent [33]. The advantages of multiplicative structures with allometric body size components against additive polynomial structures have been established [9]. In particular, in these proportional allometric models it is assumed multiplicative or proportional error ratio. Typically when plotting physiological variable such as peak VO_2_, against the body size variable, invariably it is found evidence of heteroscedasticity, that is, when the residual errors are proportional with the size of the dependent variable [9]. These characteristics in data can be dealt with the proposed log transformation. When the size variable and change trajectory (changes with exercise-based intervention) were included in the fixed part, the average size exponents were *k*′ = 0.951 (SEE = 0.137) and *k*′ = 1.075 (SEE = 0.091) for body mass and fat-free mass, respectively, which were lower than mean of interindividual coefficients. The need to allow each individual's own size exponent may be examined by letting body size descriptor vary at level 2 [4]. Due to the sample size and need to avoid over-parameterization in the models, we have only considered in the presented results (Tables 3 and 4) the variation at level 2 for the intercept and slope. Preliminary analysis suggested that the fixed parameter (mean) adequately described the population when allowing individuals to have their own size exponent. Individual variation in the changes with training trajectories was not evident, as no significant random variation was present at level 2 in all models. Moreover, between individuals variation was present at the intercept in all models (level 2 random component).

Since the present study deals with adolescent populations, important interindividual variability in body dimensions occurs during pubertal development and growth spurt needs to be considered [31]. Also, it is likely that absolute peak VO_2_ increases as a function of body dimensions during childhood and adolescence, despite individual's level of physical activity [1]. The inclusion of chronological age in the fixed model revealed a significant independent effect and reduced the average body size exponent for the sample. This suggests that older obese boys tended to show higher decrements in the peak VO_2_-body size relation with training, consistent with the trend of linear increase in peak VO_2_ with age in adolescent populations [1]. The results indicate that cardiac output and uptake of oxygen at tissue level may be more efficient in proportion to total mass and active fat-free mass in older obese boys. Also, it appears that changes in body composition, that is, proportional increase in fat-free mass in relation to the decrease of total body mass (see Table 1), as end result of the endurance training-based intervention have a beneficial to aerobic fitness and consequently in the health status of the obese boys.

Longitudinal data based on Canadian and Belgian boys indicated, on average, that peak velocity of growth in stature and peak VO_2_ tend to be coincident biological events [34]. Studies using multilevel modelling also demonstrated a size-independent effect of biological maturation on peak VO_2_ [33, 35]. Maturity-associated variation in the peak VO_2_-body size relationship during pubertal development period has been established [4, 33]. However, information dealing with the complexities of the concurrent influence of body size and biological maturation in the aerobic metabolism in response to training is limited [3]. In the present study, sexual maturity status had no significant effect in the peak VO_2_-body size relationship. However, a significant effect of the sexual maturity status indicator was observed when modeling responses in absolute peak VO_2_ to the 12-week endurance training-based intervention (interaction term age *x* maturity *β* = 0.020, 95% CL 0.009 to 0.030, *P* < 0.001). Thus, the maturity-related increases in peak VO_2_ appeared to be mediated largely by changes in body size dimensions. A limitation to the previous interpretation resides in the use of stage of sexual maturity as the indicator of biological maturity, which permits only an approximate classification of maturity, since only five discrete stages are identified [31], and given the sample size and distribution, the obese boys were grouped by PH (i.e., prepubertal versus pubertal group and postpubertal group) in the present study. Thus, more sensitive maturity indicators may need to be considered (e.g., skeletal age) in future analysis of the impact of training on obese adolescents' aerobic fitness.

## 5. Conclusions

Altogether, the results indicate that when accounting for concurrent interindividual differences in age, sexual maturity status, and body dimensions, obese boys presented a consistent improvement in peak VO_2_ in response to the 12-week exercise-based intervention. The changes in the peak VO_2_-body size relationship in response to a 12-week endurance exercise-based program in obese male adolescents were evident and at least partially influenced by chronological age. Also, the influence of interindividual variability in maturity status in peak VO_2_ responses to endurance training appears to be mediated by changes in body size. Multilevel modeling, using multiplicative model structure allows interpreting training effects over time in aerobic fitness, considering the complexities accounted to the influence of size and maturation during adolescence.

## Figures and Tables

**Table 1:** Mean changes in body size and peak VO_2_ pre- and posttraining in the obese boys (*n* = 30).

	Pretraining	Posttraining	Changes in mean 95% CL (%)	Coefficient of variation of changes in mean 95% CL (%)
Stature (cm)	158.6 (11.1)	161.0 (11.2)	1.5 (1.2 to 1.7)**	0.5 (0.4 to 0.7)
Body mass (kg)	72.4 (15.2)	70.5 (15.2)	−2.6 (−4.2 to −1.0)**	3.2 (2.5 to 4.3)
Body mass index (kg/m^2^)	28.5 (3.5)	27.0 (3.8)	−5.4 (−7.0 to −3.8)**	3.2 (2.6 to 4.4)
Body mass index (*z*-score)	3.0 (0.8)	2.5 (0.9)	−21.5 (−28.4 to −14.1)**	18.1 (14.0 to 25.3)
Waist circumference (cm)	98.9 (10.9)	96.5 (11.4)	−2.5 (−3.4 to −1.6)**	2.7 (2.3 to 3.3)
Fat-free mass (kg)	44.8 (9.7)	45.8 (9.0)	2.6 (0.6 to 4.7)**	3.9 (3.1 to 5.2)
Fat mass (kg)	26.7 (7.9)	24.9 (9.1)	−8.8 (−12.1 to −5.3)**	8.9 (7.3 to 11.5)
Peak oxygen uptake (L/min)	2.41 (0.67)	2.65 (0.65)	10.4 (4.3 to 16.4)**	12.2 (9.6 to 16.7)

*P ≤ 0.05; **P ≤ 0.01.

**Table 2:** Allometric modeling of peak VO_2_ outputs for body size variables pre- and post-exercise based intervention.

	Pretraining			Posttraining		
	Exponent	95% CL	Adjusted *R* ^2^	Exponent	95% CL	Adjusted *R* ^2^
Body mass	1.015	0.670 to 1.361	0.55	0.817	0.480 to 1.154	0.45
Fat-free mass	1.107	0.841 to 1.373	0.71	1.046	0.811 to 1.281	0.75

**Table 3:** Multilevel regression analysis of log-transformed peak VO_2_ of obese boys adjusted for body mass, age, and interaction term between age and maturity status.

	Model 1	Model 2	Model 3
	Exponent ± standard error	Exponent ± standard error	Exponent ± standard error
*Fixed explanatory variable *			
Intercept	−2.921 ± 0.632**	−2.323 ± 0.559**	−2.517 ± 0.701**
Changes with training	0.127 ± 0.030**	0.118 ± 0.030**	0.124 ± 0.030**
Body mass	0.883 ± 0.148**	0.530 ± 0.158**	0.778 ± 0.168**
Chronological age	Not entered	0.072 ± 0.019**	Not entered
Interaction age × maturity status	Not entered	Not entered	0.007 ± 0.005

*Variance-covariance matrix of random variables *			
*Level 1 (within individuals) *			
Residual	0.004 ± 0.003	0.007 ± 0.003*	0.005 ± 0.003
*Level 2 (between individuals) *			
Intercept	0.034 ± 0.010**	0.023 ± 0.008**	0.033 ± 0.010**
Changes with training	0.018 ± 0.000	0.012 ± 0.000	0.016 ± 0.000
Covariance	−0.010 ± 0.000*	−0.008 ± 0.005	−0.010 ± 0.005

−2 restricted log likelihood	−33.857	−39.489	−26.799
Akaike's information criterion	−25.857	−31.489	−18.799

*P ≤ 0.05; **P ≤ 0.01.

**Table 4:** Multilevel regression analysis of log-transformed peak VO_2_ of obese boys adjusted for fat-free mass, age, and interaction term between age and maturity status.

	Model 1	Model 2	Model 3
	Exponent ± standard error	Exponent ± standard error	Exponent ± standard error
*Fixed explanatory variable *			
Intercept	−3.159 ± 0.363**	−2.832 ± 0.389**	−3.169 ± 0.432**
Changes with training	0.076 ± 0.032*	0.081 ± 0.031**	0.076 ± 0.032*
Fat-free mass	1.058 ± 0.096**	0.860 ± 0.136**	1.061 ± 0.117**
Chronological age	Not entered	0.034 ± 0.017*	Not entered
Interaction age × maturity status	Not entered	Not entered	0.000 ± 0.003

*Variance-covariance matrix of random variables *			
*Level 1 (within individuals) *			
Residual	0.010 ± 0.004**	0.010 ± 0.004**	0.010 ± 0.004**
*Level 2 (between individuals) *			
Intercept	0.014 ± 0.006**	0.012 ± 0.005*	0.014 ± 0.006**
Changes with training	0.009 ± 0.000	0.009 ± 0.000	0.009 ± 0.000
Covariance	−0.008 ± 0.003*	−0.007 ± 0.003*	−0.008 ± 0.004*

−2 restricted log likelihood	−53.406	−53.337	−46.343
Akaike's information criterion	−49.406	−45. 337	−38.343

*P ≤ 0.05; **P ≤ 0.01.
